# Risk factors associated with intestinal parasitic infections among inmates of Kisii prison, Kisii county, Kenya

**DOI:** 10.1186/s13104-016-2191-3

**Published:** 2016-08-02

**Authors:** Dickson Cheruiyot Rop, Benson Omweri Nyanchongi, Johnson Nyangeri, Vincent Obino Orucho

**Affiliations:** Department of Biological Sciences, Kisii University, P.O. Box 408-40200, Kisii, Kenya

## Abstract

**Background:**

Intestinal parasitic infections are a major health problem worldwide. In Kenya intestinal parasitic infections are highly prevalent especially in prisons due to poor and/or inadequate infrastructure. The aim of this study was to establish the risk factors associated with intestinal parasites infection among inmates of Kisii prison, Kisii county, Kenya.

**Methods:**

Fresh stool samples of 384 inmates in Kisii prison aged 20 years and above, were screened for intestinal parasitic infections between February and August 2015. Stool samples were processed using direct fecal smear and formol-ether sedimentation techniques for confirmation then they were examined microscopically. Multiple logistic regression analysis was used to establish the relationship of various factors and practices with the occurrence of intestinal parasites. The differences were considered statistically significant at P < 0.05.

**Results and discussion:**

Of the 384 inmates screened, 95 (24.7 %) were infected with one or more intestinal parasites. Of the positive inmates, 58 (15.1 %) were infected with one species of protozoa while 24 (5.2 %) were infected with at least one species of helminthes and 13 (3.5 %) had mixed infections of species of intestinal parasites. Washing of hands before meals and after visiting toilets significantly reduced risk of infections (P < 0.05). The level of education was inversely related to the risk of intestinal parasites infection where by inmates at post primary education were least infected with intestinal parasites infection as compared to unschooled inmates (P < 0.05). Wearing of shoes was seen to significantly reduce parasitic infections (P ≤ 0.05). Duties assigned to inmates did not significantly determine the risk of intestinal parasitic infection (P > 0.05). Male inmates had significantly more intestinal parasites infections 57 (21.8 %) compared to females 28 (8.1 %) (P < 0.05). Inmates within ages 20–29 years were more infected (11.3 %) compared to the age group of >60 years (0.6 %) (P < 0.05). There was no statistical significant difference between the number of infections among the length of the jail terms (P < 0.05).

**Conclusions:**

Prevalence of intestinal parasites was high among inmates in the study area than the general population. Practices like wearing of shoes, washing of hands before meals after visiting a toilet and level of education affect the spread of the infections.

## Background

Gastrointestinal parasites represent one of the most prevalent forms of parasitic disease estimated to affect over a quarter of the world’s population [1]. Over a billion people in Sub-Saharan Africa, Asia, and the Americas are infected at any moment with at least one parasitic species; most of them leading to severe morbidity, accompanied by persistent poverty, decreased productivity, and poor socio-economic development [2].

Although intestinal parasitic infections can infect all members of a population, it is clear that there are specific groups who are at greater risk of morbidity than others, and who are more vulnerable to the harmful effects of chronic infections [3]. Among this group are inmates who carry a much burden of illness than other of society [4]. Prisoners carry a much greater burden of illness the than other members of the society this is determined both by the environment from which they come and by the prison in which they live [5]. Prisoners in developing countries live in extremely poor conditions with inadequate facilities. The prisoners are also affected by malnutrition, lack of potable water, dirty environment and very poor personal and environmental hygiene [6, 7]. Research has revealed that Kisii prisons is one of the congested prisons in Kenya with poor living conditions for the prisoners [8]. This study aimed at determining the risk factors associated with intestinal parasites, among the inmates in Kisii prison, Kisii county, Kenya.

## Methods

### Study area

The study was conducted at Kisii prison, located at Kisii town, Kisii county, Kenya between February 2015 and August 2015. The inmates came mainly from Kisii county and the neighboring counties such as Narok, Nyamira and Migori. The town is located at the southern end of the western Kenya highlands at an altitude of 1660 m above sea level. Co-ordinates for the town are 0°41′S 34°46′E/0.683°S 34.767°E. The elevation level for the town is 1700 m. It has an average daily temperature ranging from 16  to 27 °C, humidity of 88 % and average rainfall of 1500 mm per annum. This eco-climatic conditions and environmental condition influence the prevalence of infection with different types of intestinal parasites [9]. According to the 2009 Kenya census [10], the population of Kisii was estimated to be at 1,152,282 million with Kisii county making it to the list of Kenya’s most populous counties. The county has a population density of 874.7 people per km^2^ with the inhabitants being mainly civil servants, businesspersons, trader’s farmers, and casual laborers.

### Study design

This investigation involved a cross-sectional study conducted on inmates of Kisii prison. Inmates were categorized into sex, age groups, i.e., 20–29, 30–39, 40–49, 50–59 and >60 years. In addition, inmates were categorized into various jail term sentences and this comprised of those serving short-term sentences (SST) 1 year and below, serving medium term sentences (MTS) 2–5 years and those serving long-term sentences (SLT), 6 years and above. Male and female inmates who participated in the study were chosen based on their percentage population in prison. They were then requested to volunteer in the study and were randomly selected by lottery method from a list of attendance at Kisii prison dispensary for treatment.

### Study population

The study was carried out on 384 inmates (238 males and 146 female) of different jail terms of Kisii prison. This comprised of 114 awaiting trial (AWT), 187 serving jail terms (SJT), 70 serving life terms (SLT), and 10 condemned prisoners (CD) ages ranging from 20 to above 60 years that participated in this study. All the participants were informed clearly about the objective and procedure of the study and requested to sign a written consent. Research permit and ethical clearance was sought from the Kenya National Commission for Science and Technology (NACOSTI).

### Sample collection and examination

Stool specimens (about 0.5–1.5 g) were collected from 384 individuals in pre-labeled, leak-free, plastic specimen cups. The fecal specimens were physically examined and distinguished whether diarrheic (watery) or normal (well-formed samples). Diarrheic samples were examined immediately at the prison dispensary. Formed specimens were preserved in 15 ml of 10 % formalin and transported to the Kisii Teaching and Referral laboratory. The stool samples were examined by direct wet film before a confirmatory test was done using formol-ether concentration. The methods and procedures for the tests are discussed elsewhere [11]. The cysts and eggs of various parasite species present were identified. Each parasite eggs, larvae or cysts present in the samples were counted and densities of each species were expressed as “many” (>three cysts per high-power field; >20 eggs or larvae per mount); “moderate” (two cysts per high-power field; 10–19 eggs or larvae per mount); “few” (one cyst per high-power field; three to nine eggs or larvae per mount); and “rare” (two to five cysts and <two eggs or larvae per mount). For simplification, numerical values were assigned to each density: many, 4; moderate, 3; few, 2; rare, 1; and none, 0 [12].

### Ethical considerations

Institutional ethical clearance and the research permit and authorization letter were obtained from National Commission for Research Technology and Innovation (NACOSTI). Before sampling, concerned authorities such as Kenya Prison Service Department were contacted and a request for permission made after explaining the objective of the study. Prior to sample collection participants were informed clearly about the objective and procedure of the study. Participation was very voluntarily, without any slightest negative consequence, and samples were collected when they fully agreed by signing an informed consent. Potential participants were told that there were no foreseeable risk or undesirable side effect during fecal sample collection and any information obtained were to remain confidential.

### Data analysis

The data collected from the study area were entered in Microsoft office excel 2007 before being imported to SPSS version 20. Association between each exposure and the presence of infection was assessed using the Chi squared test. Logistic regression and correlation analysis was employed to determine the association between various independent risk factors and the occurrence of intestinal parasites. In all cases, P values less than 0.05 were considered statistically significant.

## Results

### Prevalence of intestinal parasites among inmates of Kisii prison

Stool specimens were collected from the inmates attending the Kisii prison dispensary. Over the 6-month period stool specimens, for 384 inmates were assayed of which 95 (24.7 %) were found to be infected with one or more intestinal parasites species. Various intestinal parasites species isolated were *E. histolytica/E. dispar*, *G. lamblia*, *B. coli*, *A. lumbricoides*, *S. stercoralis*, hookworms*, T. trichiura* and *Taenia. species.*

Fifty-eight (15.1 %) were single infections with one species of protozoan while 24 (5.2 %) were infected with one species of helminthes (Table 1). Thirteen (3.5 %) had mixed infections with various combinations where 5 (1.3 %) had *E. histolytica/E. dispar* and *G. lamblia*, 3 (0.8 %) had *E. histolytica/E. dispar* and *G. lamblia*, 2 (0.5 %) had *A. lumbricoides* and *E. histolytica/E. dispar.* In addition, *E. histolytica/E. dispar, G. lamblia and A. lumbricoides* occurred as triple infections 2 (0.5 %). *E. histolytica E. dispar* 31 (8.1 %) was the most prevalent protozoan infecting inmates while *B. coli* 4 (1.0 %) were the least prevalent protozoan. *A. lumbricoides* (4.9 %) was the most prevalent helminth infecting inmates while *T.trichiura* 1 (0.3 %) was the least helminth infecting inmates. *A. lumbricoides* and *G. lamblia* were the most prevalent parasites 5 (1.3 %) occurring as double infections. *G. lamblia,* and *Taenia* spp. 1 (0.3 %) was the least prevalent parasites occurring as double infections. The parasite score density in the faecal specimens observed ranged between 1.3 for *T. trichiura* whilst highest parasite score density was found among multiple infections with *E. histolytica/E. dispar*, *A. lumbricoides* and *G. lamblia*with 2.77.

**Table 1:** Prevalence of intestinal parasites infection among inmates of Kisii prison

Parasite class species	Number of infected cases (n)	(%) infections per species of parasites	(%) infections of those examined N = 384	Parasite density
Single infections				
Protozoa				
*E. histolytica/E. dispar*	31	53.4	8.1	2.57
*G. lamblia*	23	39.7	6	1.6
*B. coli*	4	6.9	1.0	2.3
Sub-total protozoa	58	100	15.1	
Helminthes				
*A. lumbricoides*	19	79.2	4.9	2.66
Hookworms	2	8.3	0.5	2.45
*S. stercoralis*	2	8.3	0.5	2.37
*T. trichiura*	1	4.2	0.3	1.3
Sub-total helminthes	24	100	5.2	
Mixed infections				
*A. lumbricoides* + *G. lamblia*	5	38.5	1.3	2.77
+*E. histolytica/E. dispar*	3	23.1	0.8	2.69
*A. lumbricoides* + *E. histolytica/E. dispar*	2	15.4	0.5	2.71
*A. lumbricoides* + *G. lamblia* + *E. histolytica/E. dispar*	2	15.4	0.5	2.63
*G. lamblia* + *Taenia* spp.	1	7.7	0.3	
Sub-total mixed infections	13	100	3.5	
Overall total number of infections	95		24.74	

### Temporal distribution of parasites according to the period of the study

**Fig. 1:**
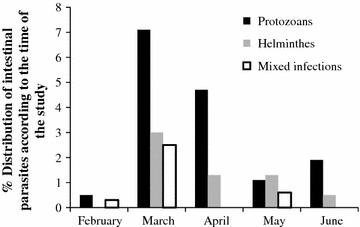
Distribution of intestinal parasites in respect to the time of the study

Intestinal parasite infections were more prevalent in the months of March, with the occurrence of 56 (12.6 %), followed by April (6.0 %), May 11 (3.0 %), June 10 (2.4 %) and February 3 (1.8 %) respectively (Fig. 1). Despite that the month of March had the highest prevalence of intestinal parasites, there were higher mixed infections in the month of April 9 (2.5 %) as compared to March 1 (0.3 %). The number of intestinal protozoan infections differed significantly with respect to the various months of the study (P < 0.05), where by the month of April 36 (7.1 %) had the highest prevalence as compared to the month of February 2 (0.5). However, the cases of intestinal helminth infections did not differ significantly over the months (P < 0.05).

### Distribution of intestinal parasites among inmates by gender

We further endeavored to establish the distribution of intestinal parasites according to genders, of which male inmates had significantly more intestinal parasites infections 57 (21.8 %) compared to females 28 (8.1 %) (P < 0.05) (Table 2). Interestingly distribution of various parasite species patterns was affected by gender, for instance *E. histolytica/E. dispar*was relatively more prevalent among males 22 (5.7 %) than female 9 (2.3 %) (P < 0,05) while *A. lumbricoides was more in* female inmates 10 (7.1 %) than their male 9 (2.3 %) counterparts. Similarly, male 19 (4.9 %) inmates were significantly more infected with *G. lamblia* as compared to females 4 (1.0 %) (P < 0.05).

**Table 2:** Distribution of intestinal parasites infections among inmates of Kisii prison by gender

% infestation rates of inmates according to gender (N = 384)			
Parasite class/species	Male (n = 238)	Female (n = 146)	(%) infestation of total
Single infestations			
Protozoa			
*E. histolytica/E. dispar*	22 (5.7)	9 (2.3)	31 (8.0)
*G. lamblia*	19 (4.9)^a^	4 (1.0)^a^	23 (5.9)
*B. coli*	2 (0.5)	2 (0.5)	4 (1.0)
Sub-total protozoa	43 (11.1)	14 (3.8)	
Helminthes			
*A. lumbricoides*	10 (7.1)	9 (2.3)	19 (9.4)
*S. stercoralis*	1 (0.4)	1 (0.4)	2 (0.8)
Hookworms	1 (0.4)	1 (0.4)	2 (0.8)
*T. trichiura*	1 (0.4)	0 (0.0)	1 (0.4)
Sub-total helminthes	4 (8.3)	11 (3.1)	
Mixed infections			
*A. lumbricoides* + *G. lamblia*	4 (1.0)	1 (0.4)	5 (1.4)
*G. lamblia* + *E. histolytica/E. dispar*	2 (0.5)	1 (0.4)	3 (0.9)
*A. lumbricoides* + *E. histolytica/E. dispar*	2 (0.5)	0 (0.0)	2 (0.5)
*G. lamblia* + *Taenia* Spp.	1 (0.4)	0 (0.0)	1 (0.4)
*A. lumbricoides* + *G. lamblia* + *E. histolytica/E. dispar*	1 (0.4)	1 (0.4)	2 (0.8)
Sub-total mixed infestations	10 (2.4)	3 (1.2)	
Overall total number of inmates	57 (21.8)	28 (8.1)	

^a^ *G. lamblia* significantly higher among males compared to females (P < 0.05)

### Distribution of intestinal parasites infections among inmates according to age groups

In this study a relatively high number of inmates infested with intestinal parasites 25 (11.3 %) was found among the 20–29 years’ age group, while the age group of >60 years was least infected with intestinal parasites 2 (0.6 %) (P < 0.05) (Table 3). Although there was no statistically significant association between age and parasitic infection, the age group 20–29 years 12 (2.5 %) had a high proportion of parasites under mixed infections compared to the age group of 30–39 years 1 (0.3 %) (P < 0.05). In addition, in all the age groups, the predominant intestinal parasite isolated was *E. histolytica/E. dispar*, followed by *G. lamblia* and *A. lumbricoides*

**Table 3:** Distribution of intestinal parasites infections among inmates of Kisii prison according to age groups

% infestation rates of inmates in respect to age groups (N = 384)						
Parasite/class	20–29 (n = 153)	30–39 (n = 124)	40–49 (n = 69)	50–59 (n = 33)	>60 (n = 5)	(%) infestation of total
Single infestations						
Protozoan’s						
*E. histolytica/E. dispar*	7 (1.8)	14 (3.6)	6 (1.7)	3 (0.8)	1 (0.3)	31 (8.2)
*G. lamblia*	1 (2.9)	6 (1.7)	4 (1.0)	2 (0.5)	0 (0.0)	23 (6.1)
*B. coli*	1 (0.3)	1 (0.3)	1 (0.3)	0 (0.0)	1 (0.3)	4 (1.2)
Sub total protozoan’s	9 (5.0)	8 (5.6)	11 (3.0)	5 (1.3)	2 (0.6)	
Helminthes						
*A. lumbricoides*	11 (2.9)^a^	6 (1.7)	2 (0.5)	0 (0.0)	0 (0.0)^a^	19 (5.1)
*S. stercoralis*	1 (0.3)	0 (0.0)	0 (0.0)	0 (0.0)	1 (0.3)	2 (0.6)
Hookworms	1 (0.3)	0 (0.0)	1 (0.3)	0 (0.0)	0 (0.0)	2 (0.6)
*T. trichiura*	1 (0.3)	0 (0.0)	0 (0.0)	0 (0.0)	0 (0.0)	1 (0.3)
Subtotal helminthes	4 (3.8)	6 (1.7)	3 (0.8)	0 (0.0)	0 (0.0)	
Mixed infections						
*A. lumbricoides* + *G. lamblia*	5 (0.7)	0 (0.0)	0 (0.0)	0 (0.0)	0 (0.0)	5 (0.7)
*G. lamblia* + *E. histolytica/E. dispar*	2 (0.5)	1 (0.3)	0 (0.0)	0 (0.0)	0 (0.0)	3 (0.8)
*A. lumbricoides* + *E. histolytica/E. dispar*	2 (0.5)	0 (0.0)	0 (0.0)	0 (0.0)	0 (0.0)	2 (0.5)
*A. lumbricoides* + *G. lamblia* + *E. histolytica/E. dispar*	2 (0.5)	0 (0.0)	0 (0.0)	0 (0.0)	0 (0.0)	2 (0.5)
*G. lamblia* + *Taenia* spp.	1 (0.3)	0 (0.0)	0 (0.0)	0 (0.0)	0 (0.0)	1 (0.3)
Subtotal mixed infections	12 (2.5)	1 (0.3)	0 (0.0)	0 (0.0)	0 (0.0)	
Overall total number of inmates	25 (11.3)	14 (8.0)	14 (3.8)	5 (1.3)	2 (0.6)	

^a^ *A. lumbricoides* significantly higher among 20–29 age groups compared to >60 age groups (P < 0.05)

### Distribution of intestinal parasites infections among inmates according to jail terms

We further investigated the distribution of intestinal parasites according to various jail terms and we found out that inmates at: short term (ST) 1 year and below, medium term (MT) 2–5 years and serving long term (SLT) 6 years and above were 32 (8.9 %), 48 (12.3 %), and 24 (6.3 %) respectively (Table 4). Inmates in medium term section were most parasitized 48 (12.3 %) compared with inmates in the long-term section who were least parasitized 24 (6.3 %). There was no statistical significant difference between the number of infections among the various jail terms (P < 0.05)

**Table 4:** Distribution of intestinal parasites infections among inmates of Kisii prison according to jail terms

% infestation rates of inmates according to prison units (N = 384)				
Parasite class/species	ST (n = 140)	MT (n = 187)	SLT (n = 83)	% infestation of total
Protozoa				
*E. histolytica/E. dispar*	10 (2.6)	15 (3.9)	6 (1.7)	31 (8.2)
*G. lamblia*	6 (1.7)	10 (2.6)	10 (2.6)	26 (6.8)
*B. coli*	1 (0.3)	3 (0.7)	4 (1.0)	8 (2.1)
Sub total protozoa	17 (4.6)	28 (7.2)	20 (5.3)	
Helminthes				
*A. lumbricoides*	9 (2.3)	7 (1.8)	3 (0.7)	19 (4.8)
*T. trichiura*	1 (0.9)	1 (0.3)	0 (0.0)	2 (1.2)
*S. stercoralis*	0 (0.0)	2 (0.5)	0 (0.0)	2 (0.5)
Hookworms	0 (0.0)	1 (0.3)	1 (0.3)	2 (0.6)
Sub total helminthes	11 (3.2)	11 (2.9)	4 (1.0)	
Mixed infections				
*A. lumbricoides* + *G. lamblia*	2 (0.5)	3 (0.7)	0 (0.0)	5 (1.2)
*A. lumbricoides* + *E. histolytica/E. dispar*	1 (0.3)	1 (0.3)	0 (0.0)	2 (0.6)
*G. lamblia* + *Taenia* spp.	0 (0.0)	3 (0.7)	0 (0.0)	3 (0.7)
*G. lamblia* + *E. histolytica/E. dispar*	0 (0.0)	3 (0.7)	0 (0.0)	3 (0.7)
*A. lumbricoides* + *G. lamblia* + *E. histolytica/E. dispar*	0 (0.0)	2 (0.5)	0 (0.0)	2 (0.5)
Sub total mixed infections	4 (1.1)	9 (2.2)	0 (0.0)	5 (1.2)
Overall total number of inmates	32 (8.9)	48 (12.3)	24 (6.3)	

*ST* short term, *MT* medium term, *LT* long term

P > 0.05

### Intestinal parasites and possible risk factors

The risk factors associated with intestinal parasites were investigated. These factors included hand wash before meals, hand wash after toilets, footwear, duties assigned to inmates and education levels

### Hand wash before meals and hand wash after visiting toilets and its association with intestinal parasites

We investigated the effects of some hygienic practices like hand washing before eating meals, and whether, they were washing their hands after visiting the toilet. Inmates who didn’t wash their hands before meals were significantly (P < 0.05) more parasitized 76 (70.4 %) compared to inmates who regularly washed hands before meals 34 (12.3 %) (Table 5). In addition, inmates who regularly washed their hands after visiting toilets were least parasitized 30 (10.9 %) than those that regularly failed to wash their hands after visiting the toilet 80 (72.1 %) (χ^2^ = 56.936, df = 1, P < 0.05).

**Table 5:** Relationship of intestinal parasites and practice of hand washing before meals and hand washing after visiting toilets

Risk factor	N	Frequency of intestinal parasites (%)	χ^2^	P
Hand washing before meals				
Yes	276	34 (12.3)	15.868	0.000
No	108	76 (70.4)		
Hand washing after toilets				
Yes	273	30 (10.9)	56.936	0.00
No	111	80 (72.1)		

### Foot wear and risk of infection with intestinal parasites

We further endeavored to investigate the effect of wearing shoes on intestinal parasite infections among the prison inmates at Kisii prison. Among inmates who had their feet not covered with shoes, 66 (64.1 %) were parasitized while 37 (35.9 %) were not parasitized. Similarly, among those who had their feet covered with shoes 46 (16.4 %) were parasitized while 235 (83.6 %) were not parasitized (Table 6). Therefore, among those inmates with intestinal parasitic infections majority 64.1 % did not wear shores as compared to 16.4 % among those who regularly wore shoes (χ^2^ = 1.106, df = 1, P < 0.05)

**Table 6:** Footwear and risk of infection with intestinal parasites

Risk factor	N	Frequency of intestinal parasites (%)	χ^2^	P
Wearing of shoes				
Yes	281	46 (16.4)	1.106	0.00
No	103	66 (64.1)		

### Duties assigned to inmates and its association with intestinal parasites

In the study we further sought to know whether the duties assigned to the inmates was associated with the risk of intestinal parasite infections. Among the inmates, 268 (69.8 %) were artisans, 100 (25 %) were farmers and 26 (4.2 %) were cooks (Table 7). Although there was no statistically significant association between duties assigned to inmates and parasitic infection, (χ^2^ = 0.832, df = 2, P < 0.05), farmers 82 (30.6 %) had a relatively high percentage of parasitic infection compared to artisans 25 (25 %).

**Table 7:** Relationship of intestinal parasites and duties assigned to inmates

Risk factor	N	Frequency of intestinal parasites (%)	χ^2^	P
Duties of inmates				
Artisan	100	25 (25)	0.082	0.484
Farmers	268	82 (30.6)		
Cooks	26	5 (19.2)		

### Education levels and its association with intestinal parasites

Additionally, we evaluated the effect of education that is commonly associated with awareness about parasitic infection risks. Prisoners who reported to have been in post primary institution had low prevalence of intestinal parasites 17 (21.5 %) whereas those inmates who reported to have not gone to school had high prevalence of intestinal parasites 31 (37.4 %) (Table 8). The level of education of study participants was significantly associated with intestinal parasitic infections (χ^2^ = 5.260, df = 3, P < 0.05)

**Table 8:** Relationship of intestinal parasites and education levels of inmates

Risk factor	N	Frequency of intestinal parasites (%)	χ^2^	P
Education level				
Unschooled	83	31 (37.4)	0.733	0.070
Primary	171	53 (30.9)		
Post primary	130	17 (21.5)		

## Discussions

In this study, 24.7 % of the prisoners were infected with one or more species of intestinal parasites. This rate was higher than the rate reported by Kamau et al. [13] (15 %) who carried out a study on the prevalence of intestinal parasitic infections in non-prisoners certified food- handlers working in food establishments in Kenya. More infections were seen in the male prisoners especially those with poor hygienic practices because of irregular washing of hands before meals or after visiting the toilet and irregular wearing of shoes. Conversely, this infection was found to be higher than the rate reported by similar studies among inmates in Nigeria [14, 15] that found prevalence rates of 22.8 and 9.0 % respectively. However, the findings of the current study were lower than the rate reported by Rasha et al. [16] in Ethiopia (49 %). The current study also found, the prevalence rate of helminth infection (5.2 %) was found to be lower than that of protozoan infection (15.1 %). This concurred with the work of Okolie [17] who reported a prevalence of protozoan infection (32.40 %) as compared to helminth infection (22.40 %) but contrasted from earlier studies by [18], who found helminthes as the highest (59.8 %) as compared to (42.3 %) for protozoan’s. This is because protozoa are immediately infectious, where eggs of helminthes may need a while to become infective [18].

Of the 384 inmates examined, *E. histolytica/E. dispar*was the most prevalent intestinal parasite accounting for 31 (8.1 %) of inmate infections. The detection of *E. histolytica* in stool samples examined even at low prevalence is a serious concern for health hazard free environment and poses a risk to public health; this is because *E. histolytica* is more pathogenic [19]. The higher rate of feco-orally transmitted infections like *A. lumbricoides, G. lamblia,* and *E. histolytica/E. dispar* indicates dissemination of these infections under institutional conditions. However, *A. lumbricoides* and *E. histolytica/E. dispar* differ a bit in their transmission. Although both orally, protozoa are immediately infectious, whereas eggs of *Ascaris* spp may need a while to become infective. Because of this reason, autoinfection may be common for *E. histolytica/E. dispar* [18]. Once infected, individuals may indefinitely propagate the protozoa unless treated. In addition, even if hygienic facilities improve in the institution it might be impossible to clean the participants from preexisting infection, especially *Entamoeba* spp. This may explain the most common occurrence of *E. histolytica/E. dispar* among the study populations in the present study. Poor hygiene practices have been increasingly recognized as a major route of transmission of *E. histolytica/E. dispar*worldwide [2]. The higher prevalence of *A. lumbricoides* than hookworms*’* prevalence was contrast to a previous report from eastern Nigeria Ohaegbula and Hassan [20, 21], who reported that *A. duodenale*occurs more frequently in mild and humid conditions as the free-living larval stages are unlikely to survive under extremes of temperature and desiccation. Similarly, the higher prevalence of *A. lumbricoides* 20 (4.8 %) could have been due to eating of raw or undercooked vegetables or unwashed fruits among the inmates. The presence of moderate parasite score densities among the inmates indicate high transmission risks of such parasites to inmates of Kisii prison. These findings indicate a public health priority and strongly support the need for improvement of sanitation conditions in and around the prison in order to reduce the prevalence of infection among the inmates.

In addition, the levels of infection were higher during the months of March 56 (12.2 %) and April 24 (6.0 %) as compared other months during the study. This was especially true for the protozoans that reproduce and show pathology within a short time span. This changes could be attributed to the weather changes experienced in the region. Kisii has two rain seasons between March and May and Aug–Nov. These findings were consistent with observations by Mahfouz et al. [9] that parasitic infections were directly related to the pattern of rainfall. The number of intestinal parasites infections differed significantly with respect to the various months of the study P < 0.05, where by the month of April 56 (12.6 %) had the highest prevalence as compared to the month of February 3 (1.8 %).

We endeavored to establish the distribution of intestinal parasites among male and female inmates and we observed that the former had significantly higher prevalence (P < 0.05) of intestinal parasites infection than the latter 57 (21.8 %) and 28 (8.1 %), respectively. This is in contrast with a study by Mamman and Reuben [15] who reported comparable percentage prevalence of intestinal parasites among males (48.7 %) and females (49.3 %). This could be because female inmates have better personal hygiene practices, as findings from the results showed that males 124 (73.3 %) who practiced hand-washing practice was lower as compared to females 184 (84.9 %) who practiced hand washing regularly. The rates among males and females may have differed due to overcrowding, where there was an average of 56 inmates per cell for males and 16 inmates per cell for females. The male cells were designed to house and average of 25 inmates while the female cells were designed to house an average of six inmates. Previous studies have shown that an overcrowded urban environment promotes the prevalence of intestinal parasites infections, for instance Atukurola et al. [22] observed that crowding enhances the transmission of intestinal parasitic infections. Moreover, male inmates have been reported to be practicing oral anal sex [8], thus a need for investigations to establish whether it contributes to these high infections.

We further investigated distribution of intestinal parasites infection with respect to various age groups using the Pearson correlation and we found out that there was a negative correlation coefficient (P = 0.83) 2-tailed, which means that as age increases the burden of intestinal parasites infection reduces. Inmates belonging to age groups of 20–29 years 25 (11.3 %) had significantly higher prevalence of intestinal parasites infection compared to the age group of >60 years 2 (0.6 %) (P < 0.05). These findings are similar to those by Rasha et al. [16] who reported a prevalence of 25 (9.1 %) among age groups of 21–30 years. These age groups are highly infected groups in the prison probably because they are the most active thus high exposure to the parasites, increasing their risk of infection. The age group of >60 years had the lowest prevalence of 2 (0.6 %) which concurring with an investigation by Colman et al. [14] who reported lower prevalence in the older ages. This might be because these individuals are confined and thus their movements are restricted. This study also agrees with the work of Okolie [17], who reported high intestinal parasites infections among young adults’ inmates of Owerri prison thus this age group should be prioritized when conducting any prevention strategies.

In the study, we further sought to know whether the distribution of intestinal parasitic infections was based on the various jail terms, inmates in serving short term section (SST) 48 (12.3 %) had significantly high prevalence of intestinal parasitic infections as compared to the inmates in serving long term section 32 (8.9 %) (P < 0.05). This finding concurred with the reports by Mamman and Reuben [14] who reported a higher prevalence of (14 %). This is because inmates in this unit (short-term section) are regarded as the mobile and working inmates. They were often taken out for manual jobs and this predisposed them to intestinal parasites infections. Low intensity of intestinal parasites infection was recorded among inmates at long-term section 32 (8.9 %). This was due to their constant confinement, which prevents them from being exposed to intestinal parasitic organisms.

The effects of hygiene practices were investigated including whether the inmates regularly washed hands before meals or after visiting toilets. Such practices promote the ingestion of most of the viable and infective intestinal protozoan cysts (*E. histolytica/E. dispar* and *G. lamblia*) and infective embryonated nematode ova (eggs) of *A. lumbricoides* and *T. trichiura*. Indeed, in some cases, infective larvae of *A. duodenale* and *S. stercoralis* present in contaminated, raw, or inadequately cooked foods and drinks. Correlation analysis showed that there was significant difference between inmates who washed hands before meals 76 (70.4 %) compared to those who did not washed their hands before meals 34 (12.3 %) (P < 0.05). Moreover, there was significant difference between inmates who did not washed their hands before visiting toilets 80 (72.1 %) as compared to those who washed their hands after visiting toilet 32 (8.9 %) (p < 0.05). This means that poor hand washing practices is associated with intestinal parasites. For example, most of the inmate used flush water method of fecal disposal, which had inadequate supply of water and this could contribute widely to the transmission of intestinal parasites among the inmates especially when there is long accumulation of faces. This is particularly the case with the Kisii prisons as the flush toilets used by the inmates have poor flushing facility, poorly maintained toilets that mostly lack piped water and broken sewage pipes increased chances of mechanical transmission of cysts and eggs of intestinal parasite among the inmates. Toilets, which were not frequently cleaned, invited the breeding of toilet flies (*Fannia scalaris*) which transmit intestinal parasites infections mechanically [23].

In this study, we further sought to know whether the duties assigned to the inmates was associated with the risk of intestinal parasite infections, where by intestinal parasitic infections were not significantly associated with the duties assigned to inmates in prison P > 0.05. However, intestinal parasitic infections varied differently according to various duties assigned to inmates, whereby farmers 82 (30.6 %) had high infection as compared to artisans 25 (25 %) and cooks 5 (18.8 %). This finding contrast with the finding conducted at Owerri prison by Okolie [17] who reported duties assigned to inmates as being significant.

Additionally, we evaluated the effect of the levels of education on the risk of infection with intestinal parasites. Higher level of education is usually associated with awareness about parasitic infection risks and this was observed in this study, where unschooled inmates were mostly infected with intestinal parasites 31 (37.4 %) as compared to those inmates who reported to have been in post primary education 17 (21.5 %) P < 0.05. This observation is similar to a study by Aschalew et al. [24] at Zarima town in Ethiopia that reported an association between infection by parasites and levels of education.

We further endeavored to investigate the effect of wearing shoes on intestinal parasite infections among the prison inmates at Kisii prison. The low prevalence of hookworms than *A. lumbricoides* could be attributed to the fact that most of the subjects had their feet covered with shoes thus could not be exposed to these intestinal parasites. The major and common portal of entry of some helminthes is the skin of the foot. Inmates who did not wear shoes had higher intestinal parasite infections 66 (64.1 %) as compared to those who did not wear shoes 46 (16.4 %) (P < 0.05). The number of infected prisoners with hookworms was small, this could be positively attributed to reduced exposure to the parasite. Given that there were prisoners walking bare feet, more infections are likely to be seen with time. This study conforms to a similar study by Wekesa et al. [25] who observed that among risk factors contributing to infections by intestinal parasites to pregnant women were irregular wearing of shoes which ranked second highest (88.5 %). The prison authorities should consider providing shoes to inmates to curb transmission of geohelminthes.

## Conclusions

Intestinal infections within the Kisii prisons are higher than the general population with *Entamoeba histolytica* and *A. lumbricoides* being the most prevalent protozoa and helminthes respectively among the inmates. *Balantidium coli* and *Trichuris trichiura* were the least protozoan’s and helminthes respectively among the inmates. Education levels, health practices including hand washing before meals, eating habits, foot wear and general personal hygiene contributes to intestinal parasitic infections.

## Abbreviations

KTRH: Kisii Teaching and Referral hospital
LT: long-term sentences
MTS: medium term sentences
NACOSTI: National Commission of Science and Technology
NTDS: neglected tropical diseases
ST: short term
SPSS: Statistical Package for Social Scientists
